# Factors associated with ocular prosthesis motility in anophthalmic patients: a multivariable analysis

**DOI:** 10.1007/s10792-026-04320-3

**Published:** 2026-11-10

**Authors:** Yoav Vardizer, Oren Tomkins-Netzer, Yinon Shapira

**Affiliations:** 1 The Ruth and Bruch Rappaport Faculty of Medicine, Technion ‑ Israel Institute of Technology, Haifa, Israel; 2 Department of Ophthalmology, Bnai Zion Medical Center, Haifa, Israel; 3 Department of Ophthalmology, Carmel Medical Center, Haifa, Israel

**Keywords:** Ocular prosthesis, Artificial eye, Anophthalmic socket, Prosthesis motility, Cosmetic rehabilitation, Cosmetic appearance, Motility assessment

## Abstract

**Purpose:**

To identify the clinical, anatomical, and prosthetic factors independently associated with ocular prosthesis motility in anophthalmic patients.

**Methods:**

This prospective cross-sectional study included 107 patients with unilateral anophthalmia wearing acrylic prostheses. Horizontal prosthesis motility was graded relative to the fellow eye by three independent masked examiners using a five-point ordinal scale. Candidate predictors associated in univariate analysis (p ≤ 0.10) were entered into blockwise multivariable regression across five domains: clinical background, socket and eyelid findings, socket motility parameters, prosthesis primary gaze alignment, and prosthesis structural dimensions.

**Results:**

Independent predictors of prosthesis motility were socket temporal motility (β = 0.36, p < 0.001), time since anophthalmia (β = –0.23, p = 0.003), prosthesis primary gaze alignment (β = 0.21, p = 0.002), socket downward motility (β = 0.20, p = 0.027), inferior fornix depth (β = 0.19, p = 0.007), and levator function (β = 0.15, p = 0.042), together explaining 67% of the variance in examiner-rated prosthesis motility (adjusted R^2^ = 0.64). Prosthesis dimensions, demographic variables, and orbital implant status did not retain independent significance.

**Conclusion:**

In this study, prosthesis motility was primarily associated with socket dynamics, structural socket capacity, eyelid function, and chronicity of anophthalmia. These findings highlight the importance of multifactorial clinical assessment when evaluating prosthetic eye movement as part of visual and cosmetic rehabilitation after eye loss.

**Supplementary Information:**

The online version contains supplementary material available at https://doi.org/10.1007/s10792-026-04320-3.

## Key points

- Prosthesis motility is a major determinant of cosmetic satisfaction after eye loss.
- Prior studies have focused mainly on implant type, surgical technique, and coupling strategies, with limited evaluation of broader anatomical contributors.
- In this multivariable analysis, prosthesis motility was independently associated with socket motility, inferior fornix depth, levator function, prosthesis alignment, and chronicity of anophthalmia.
- These findings suggest that visible prosthesis excursion reflects broader socket biomechanics rather than implant-related factors alone.

**Supplementary Information:**

The online version contains supplementary material available at https://doi.org/10.1007/s10792-026-04320-3.

## Introduction

Cosmetic appearance is a central component of rehabilitation after eye loss, and prosthesis motility plays a key role in the perceived naturalness of the artificial eye during everyday social interactions and observer perception. Prior studies show that reduced motility is a primary source of concern for prosthesis wearers, who are often more affected by how they appear to others than by their own self-perception [1–4]. Patient-perceived motility is a strong predictor of satisfaction with the overall appearance of the artificial eye, and observer-rated motility similarly correlates with externally assessed cosmetic outcomes, underscoring its central role in cosmetic rehabilitation and overall well-being [5, 6].

Substantial clinical and surgical effort has therefore been directed toward improving motility through refinements in implant design and coupling, surgical technique, and socket anatomy optimization [7–9]. Despite decades of practice, empirical evidence regarding which measurable clinical or anatomical features most strongly influence prosthesis motility, and how best to assess them clinically, remains limited [3].

This study aimed to identify the clinical, anatomical, and prosthetic factors independently associated with examiner-rated prosthesis motility in anophthalmic patients using multivariable models integrating background, socket, eyelid, and prosthesis characteristics.

## Methods

### Study design and participants

This was a prospective, cross-sectional study conducted at a tertiary ocular prosthetics clinic. Patients were identified prospectively during routine follow-up visits at the ocular prosthetics clinic, and consecutive eligible adult patients with unilateral anophthalmia were invited to participate. Eligibility criteria included age 18 years or older, use of a unilateral custom-made acrylic (PMMA) ocular prosthesis, and ability to complete a self-administered questionnaire. All prostheses were fabricated using an impression-moulding technique by the same ocularist (Y.V.) at the referral ocularist center. Patients with bilateral or congenital anophthalmia, those wearing cosmetic scleral shells, or individuals unable to complete study procedures were excluded. Institutional Review Board approval was obtained (Approval No. 0023–09-BNZ), and written informed consent was obtained from all participants before enrollment.

## Clinical assessment

Each participant underwent a structured ophthalmic and prosthetic examination conducted by three independent, masked examiners—an oculoplastic surgeon, an ocularist, and a senior ophthalmologist. The assessment included demographic and clinical background information, socket and eyelid parameters, and prosthesis characteristics. Socket and eyelid findings comprised marginal reflex distance−1 (MRD1), levator function, lagophthalmos on gentle closure, lower-lid laxity, relative exophthalmometry defined as the difference in anterior projection between the prosthetic and fellow sides (prosthetic minus fellow eye), socket support contour (clinically categorized as relatively rounded or flat), conjunctival contracture, and the depths of the superior and inferior fornices. Fornix depth was measured with the corresponding eyelid gently pulled forward from the lid margin, and a ruler was used to measure the distance from the eyelid margin to the transition of the conjunctival cul-de-sac to the bulbar conjunctiva. Orbital implant status was established from the clinical record and/or clinical examination and was documented for all participants. The alignment of the prosthetic iris in primary gaze relative to the fellow eye was assessed.

Directional socket motility was assessed clinically and independently by the examiners in each direction of gaze, without a fixed reference mark, and was evaluated with the prosthesis removed. Patients were asked to follow the examiner’s finger with their gaze in horizontal and vertical directions, and the resulting displacement of the conjunctival surface was graded in each direction (nasal, temporal, upward, and downward) as poor, mediocre, or good. An overall impression of socket motility was also recorded.

Prosthesis dimensions were evaluated after removal. Volume was measured by water displacement, and caliper measurements were used to determine central thickness and maximal posterior apex height.

## Outcome measure

**Fig. 1:**
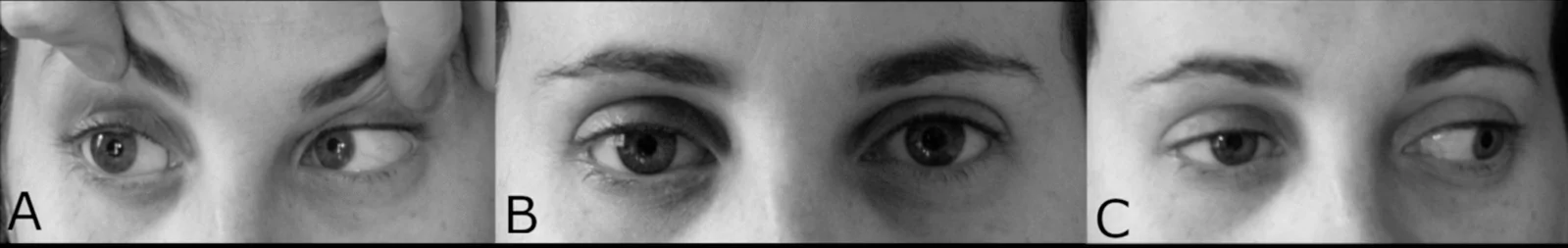
Example of examiner-rated ocular prosthesis motility. Standardized photographs demonstrate horizontal gaze excursion in a participant with grade 4 prosthesis motility, defined as more than 50% of the fellow eye’s excursion. **A** Right gaze, **B** primary gaze, demonstrating alignment of the prosthetic iris with the fellow eye, and **C** left gaze

The primary outcome was examiner-rated prosthesis motility as a pragmatic clinical measure of visible prosthetic eye movement. With the prosthesis in place, standardized photographs were obtained in primary gaze and at the horizontal gaze extremes, with patients instructed to follow the examiner’s moving finger through the full horizontal amplitude of gaze (Fig. 1). Three examiners independently reviewed the photographs and graded the horizontal amplitude of prosthesis movement relative to the fellow eye on a five-point ordinal scale: 1 = minimal or none, 2 = less than 50% of the fellow eye’s excursion, 3 = approximately 50%, 4 = more than 50%, and 5 = almost full or near-normal excursion. The average of the three examiner scores was used for all analyses after verifying inter-rater reliability.

## Statistical analysis

Inter-rater agreement was assessed using Kendall’s coefficient of concordance. Associations between prosthesis motility and potential predictors were examined in univariate analyses using Spearman’s rank correlation for continuous variables and the Mann–Whitney U test or the Kruskal–Wallis test for categorical variables.

To address the large number of potential predictors and the risk of collinearity, a blockwise multiple regression approach was employed. Blockwise modeling was chosen a priori to accommodate a relatively large set of conceptually distinct predictors within a moderate sample size and to reduce multicollinearity by structuring variables into clinically coherent domains [10]. Variables associated with prosthesis motility in univariate analysis (p ≤ 0.10) were entered into domain-specific multiple linear regressions for the five predefined blocks: (A) clinical background, (B) socket and eyelid findings, (C) socket motility parameters, (D) prosthesis primary gaze alignment, and (E) prosthesis structural dimensions.

Within each block, variables that remained independently associated with prosthesis motility (p < 0.05) were retained. These retained variables from all five blockwise models were then pooled and entered simultaneously into a final integrated multivariable linear regression model.

Model performance was expressed as the coefficient of determination (R^2^) and adjusted R^2^ for each blockwise model and for the final integrated model. Standardized regression coefficients (β) from the final integrated model were used to indicate the relative independent contribution of each retained predictor to examiner-rated prosthesis motility. Statistical significance was defined as p < 0.05. All analyses were performed using Statistica software, version 12 (StatSoft Inc., Tulsa, OK, USA).

## Results

The inter-rater correlations for motility ratings were moderate to good (r = 0.49–0.63, p < 0.001); therefore, mean examiner scores were used for analyses. Clinical, anatomical, and prosthesis-related data are summarized in Supplementary Table 1. The univariate correlations between examiner-rated prosthesis motility and potential predictors are shown in Table 1.

**Table 1:** Univariate analysis of the association of clinical, eyelid, socket, and prosthesis-related parameters with examiner-rated prosthesis motility scores, in 107 anophthalmic patients with an artificial eye

		Motility score	
	Variable		P-value
Demographics / Clinical Background	Age (Years)	−0.21	0.034
	Gender		0.25
	Country of birth		0.46
	Religion		0.72
	General health		0.37
	Eye		0.89
	Cause of anophthalmia		0.077
	Time with anophthalmia (Years)	−0.40	< 0.0001
	Orbital implant present		< 0.0001
	Additional periorbital surgeries		0.43
Socket and Eyelid	MRD1 (vs. fellow eye, mm)	0.23	0.024
	Levator function (mm)	0.53	< 0.0001
	Lagophthalmos (mm) comb	−0.48	< 0.0001
	Lower eyelid laxity		0.097
	Exophthalmometry (vs. fellow eye, mm)	0.06	0.55
	Socket support		< 0.0001
	Inferior fornix depth (mm)	0.35	< 0.001
	Superior fornix depth (mm)	0.26	0.010
	Inferior fornix position		0.006
	Socket contracture	0.47	< 0.0001
Socket motility	Down	0.63	< 0.0001
	Nasal	0.53	< 0.0001
	Up	0.52	< 0.0001
	Temporal	0.60	< 0.0001
	General impression	0.67	< 0.0001
Prosthesis alignment	Prosthesis primary gaze alignment		0.018
Prosthesis Structural dimensions	Volume (ml)	0.07	0.48
	Central thickness (T, mm)	−0.34	< 0.001
	Posterior apex height (D, mm)	0.50	< 0.0001
	Posterior projection index (D/T)	0.47	< 0.0001

In the blockwise regression models (Table 2, Supplementary Table 2), the explanatory power for the motility ratings (R^2^) was 0.56 for socket and eyelid findings, 0.54 for socket motility ratings, 0.36 for clinical background variables, 0.15 for prosthesis structural dimensions, and 0.06 for prosthesis primary gaze alignment. In the final integrated regression model (Table 2, Supplementary Table 2), the retained variables from the separate blockwise models were entered simultaneously, and the independent predictors of prosthesis motility (in descending order of standardized β coefficients) were socket temporal motility (β = 0.36, p < 0.001), time since anophthalmia (β = –0.23, p = 0.003), inferior fornix depth (β = 0.19, p = 0.007), prosthesis primary gaze alignment (β = 0.21, p = 0.002), socket downward motility (β = 0.20, p = 0.027), and levator function (β = 0.15, p = 0.042). The final model explained 67% of the variance in examiner-rated prosthesis motility (adjusted R^2^ = 0.64).

**Table 2:** Summary of blockwise multiple regression analysis for predictors of prosthesis motility in 107 anophthalmic patients with an artificial eye

Block	Variables tested^1^	Variance in examiner-rated prosthesis motility explained by each block regression model (R^2^)^3^
A. Clinical background	Age, **Time with anophthalmia**, Cause of anophthalmia, **Orbital implant**	0.36
B. Socket and eyelid	MRD1 (relative), **Levator function**, Lagophthalmos, Lower eyelid laxity, **Socket support**, **Inferior fornix depth**, Superior fornix depth, Inferior fornix position, Socket contracture	0.56
C. Socket motility	**Down**, Up, Nasal, **Temporal**, General impression	0.54
D. Prosthesis alignment	**Prosthesis primary gaze alignment**	0.06
E. Structural dimensions	Central thickness, Posterior apex height, Posterior projection index	0.15
*Integrated model (final)*^2^	**Time with anophthalmia**, Orbital implant, **Levator function**, Socket support, **Inferior fornix depth**, **Socket downward motility**, **Socket temporal motility**, **Prosthesis primary gaze alignment**	*0.67*

^1^Variables with p ≤ 0.10 in the univariate analysis were entered in the domain-specific blockwise multiple regression modeling

^2^Variables that remained significant (p < 0.05) within the separate blockwise models were pooled and entered simultaneously into the final integrated multivariable model

^3^R^2^ values represent the proportion of variance in examiner-rated prosthesis motility explained by each individual block model or by the final integrated model

Values in **boldface** mark a significant independent predictive effect of the parameter (< 0.05) in the respective regression.

Although orbital implant status showed significance within its block, it was not retained in the final integrated model. Orbital implant presence was significantly associated with greater socket motility in all directions (downward 2.28 ± 0.08 vs 1.53 ± 0.10, nasal 1.97 ± 0.08 vs 1.47 ± 0.09, upward 2.38 ± 0.10 vs 1.76 ± 0.11, and temporal 2.33 ± 0.09 vs 1.73 ± 0.11; all p < 0.001).

Conjunctival contracture, despite showing a notable univariate association with prosthesis motility, did not retain significance in the regression analysis. This may be due to its strong correlation with inferior fornix depth (r = 0.43, p < 0.0001), which itself remained an independent predictor in the final model.

Socket support contour was significantly associated with prosthesis motility within the socket and eyelid block regression, but lost significance in the final integrated model. Rounded sockets showed greater inferior fornix depth (8.2 ± 0.3 mm vs 7.0 ± 0.4 mm, p < 0.001) and less conjunctival contracture (mean rating 2.9 ± 0.1 vs 2.6 ± 0.1, p = 0.02).

**Fig. 2:**
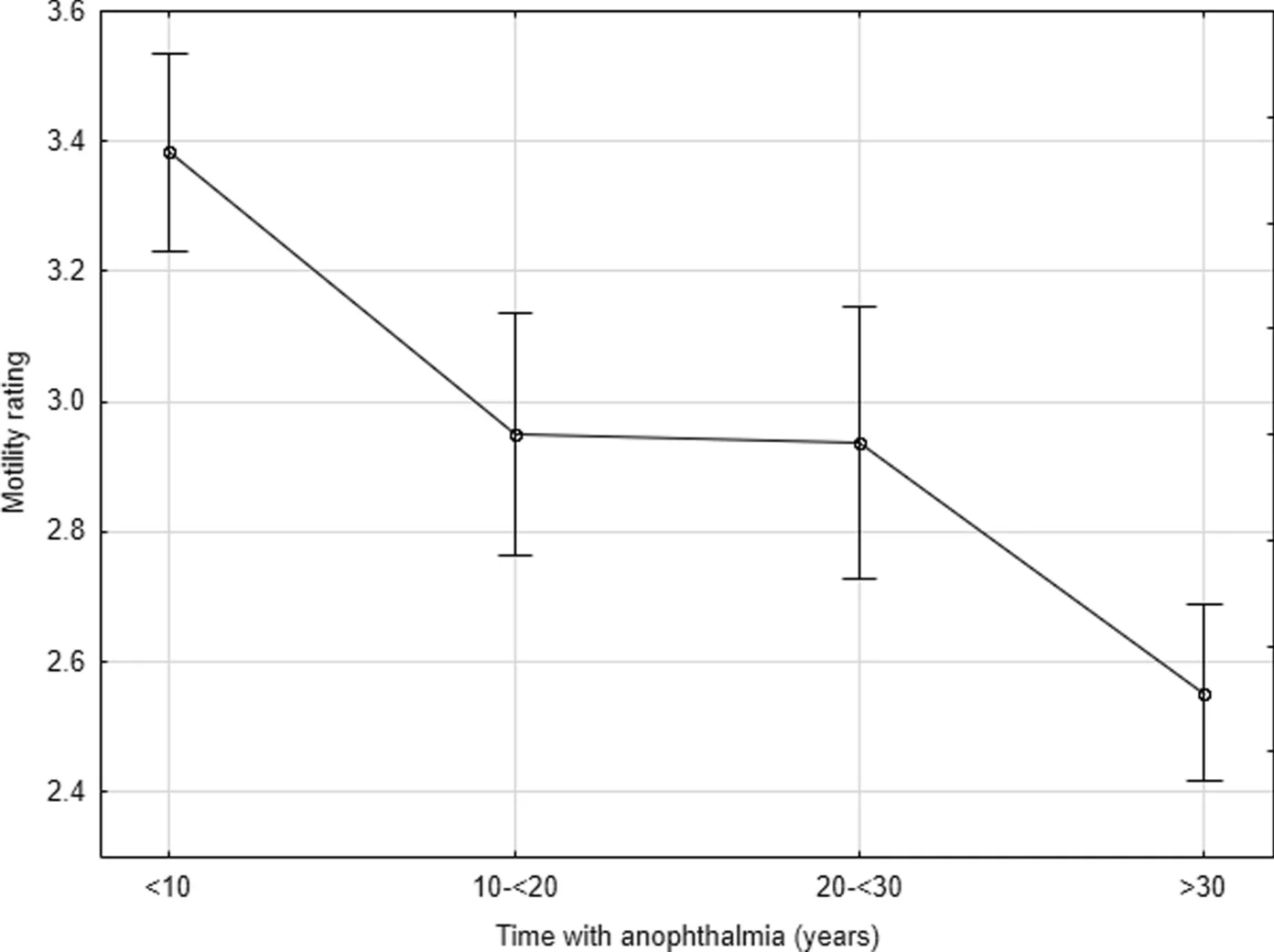
Mean prosthesis motility scores (± SD) across four anophthalmia-duration categories (< 10, 10–< 20, 20–< 30, and ≥ 30 years). Motility declined progressively with increasing duration (p < 0.001, Kruskal–Wallis test)

Finally, the time since anophthalmia showed an inverse association with prosthesis motility (Table 2, Fig. 2). Mean motility scores declined progressively with longer duration of anophthalmia: 3.38 ± 0.15 for less than 10 years (n = 30), 2.95 ± 0.19 for 10 to less than 20 years (n = 20), 2.94 ± 0.21 for 20 to less than 30 years (n = 16), and 2.55 ± 0.14 for 30 years and more (n = 38; p < 0.001). This association remained significant after adjustment for age, which itself did not retain significance in the regression.

## Discussion

Prosthesis motility is widely recognized as central to cosmetic and psychosocial rehabilitation after eye loss, and substantial effort has therefore been directed toward optimizing movement of the artificial eye. Traditionally, these efforts have focused on implant material and design, surgical technique, and direct coupling mechanisms [7, 8, 11, 12]. However, robust comparative evidence supporting these strategies remains limited. Direct comparisons between porous and non-porous implants have demonstrated similar motility when surgical technique is standardized [7, 12], while high-quality comparative data between implant designs remain limited [13]. Differences reported between enucleation and evisceration [14] may diminish with optimized extraocular muscle reattachment techniques [15]. While pegging can enhance horizontal motility, it carries a risk of complications and is now infrequently used [9, 16, 17].

Against this background, there has been limited systematic evaluation of the broader anatomical and biomechanical factors associated with prosthesis movement. The present multivariable modelling identified a set of factors independently associated with horizontal prosthesis motility. Directional socket motility (especially temporal), chronicity of anophthalmia, inferior fornix depth, primary gaze alignment, and levator function each retained independent associations and together explained a substantial proportion of motility variability.

Directional socket motility showed the strongest independent association with horizontal prosthesis excursion, supporting the principle that visible prosthetic movement depends on the socket’s capacity to generate and transmit excursion. Nakra et al. [14] demonstrated that implant motility exceeds prosthesis motility, and Bigheti et al. [11] further showed that movement amplitude decreases when the external prosthesis is in place, underscoring that force transmission from implant to prosthesis is inherently attenuated. Implant presence has been shown to influence prosthetic motility [3] and, in our cohort, was strongly associated with greater socket motility in all directions. However, implant status did not retain an independent association with prosthesis motility in the integrated model once socket motility and other retained factors were considered. This suggests that the contribution of an orbital implant to visible prosthesis movement may be expressed, at least in part, through the socket movement it generates.

Inferior fornix depth was independently associated with horizontal prosthesis motility. Although most commonly discussed in relation to prosthesis retention, adequate inferior fornix depth is essential for stable seating and proper apposition of the prosthesis [11, 18]. In our multivariable model, inferior fornix depth retained independent significance, whereas socket support contour and conjunctival contracture did not. This suggests that fornix depth may function as an integrative structural parameter, capturing several interrelated features such as available conjunctival surface area, cul-de-sac capacity, and prosthesis apposition that collectively influence coupling efficiency. Importantly, dynamic socket movement can translate into visible prosthetic excursion only when sufficient fornix depth permits mechanical excursion without restriction, underscoring the interdependence of structural capacity and socket motility.

Duration of anophthalmia was independently associated with prosthesis motility, whereas chronological age did not retain significance in the multivariable model. Van Renterghem et al. [3] reported worse abduction in patients who underwent eye removal at a younger age and discussed age-related decline in implant motility, echoing similar earlier findings by Custer et al. [12]. Because age and duration of anophthalmia are closely interrelated, their simultaneous inclusion in the multivariable model enabled assessment of their independent associations with prosthesis motility; and only duration retained significance. This suggests that duration of anophthalmia may be more closely associated with reduced prosthesis motility than chronological age itself, although the cross-sectional design does not allow the underlying mechanism to be determined.

In contrast, patient-reported outcome studies indicate that perceived concerns about appearance and motility tend to stabilize or diminish with older age and longer duration of the anophthalmic condition [5, 19]. This divergence likely reflects psychosocial adaptation over time rather than true objective improvement in prosthesis movement.

Levator function demonstrated a modest but independent association with prosthesis motility in the integrated model. Although its effect size was smaller than that of socket motility or fornix depth, its persistence suggests that eyelid dynamics may influence the visible amplitude of horizontal excursion. Anophthalmic alterations in orbital volume and implant positioning have been shown to affect the levator pivot point and reduce levator efficiency, while restoration of anterior projection can improve eyelid function [20]. Decreased levator function has also been documented in the anophthalmic orbit, independent of overt ptosis [21]. In this context, preserved levator function may reflect more favorable periocular muscle balance and anterior socket configuration, contributing modestly to the observer-rated impression of movement.

From a clinical perspective, several of the factors identified may be modifiable. Good socket motility may be supported by appropriate implant size and position, preservation or reattachment of the extraocular muscles, and meticulous primary surgical technique; in patients without an implant, secondary implantation represents a potential reconstructive option for restoring orbital volume and supporting socket movement. Likewise, socket contraction and inadequate fornix depth may be amenable to reconstructive procedures aimed at restoring socket capacity and prosthesis support. The association with levator function may partly reflect implant position and orbital volume, which can alter periocular muscle vectors and eyelid mechanics, while prosthesis alignment can be optimized during ocularist fitting. These findings therefore broaden the surgical implications beyond implant selection alone and suggest several potential targets for primary surgery, secondary reconstruction, and prosthetic rehabilitation.

Finally, the measurement of prosthesis motility remains methodologically challenging. The 2018 American Academy of Ophthalmology Ophthalmic Technology Assessment noted that studies assessing motility were “not uniformly based on objective analysis” [7], and the 2016 Cochrane review similarly highlighted substantial clinical heterogeneity and variability in measurement approaches, limiting direct comparisons across studies [13]. Most investigations assess maximal static excursion in extreme gaze positions, whereas parameters such as saccadic velocity or pursuit characteristics—standard in strabismus evaluation—have not been systematically applied to prosthesis motility assessment (Table 3). Subtle low-amplitude movements near primary position, which may be more relevant to natural appearance during conversation and social disguisability, are not usually captured [11]. Our semi-quantitative ordinal grading shares the limitations of static excursion-based methods and may be influenced by observer interpretation; moreover, such scales are not standardized across studies, complicating external comparison. However, the use of three independent masked raters and a predefined ordinal scale enhances internal consistency.

**Table 3:** Ocular prosthesis motility assessment methods

Method	Motion type	Output units	Advantages	Limitations	Reference
Ruler measurement	Full amplitude	mm	Simple, inexpensive, widely available	Subjective endpoint determination; inter-observer variability; requires patient cooperation	[22]
Kestenbaum limbus test	Full amplitude	mm	Simple, portable	Requires visible limbus marking on prosthesis; inter-observer variability	[3]
Slit-lamp device	Full amplitude	mm	Objective measurement; real-time video documentation; reproducible	Requires custom device; not commercially available; equipment cost	[15]
Magnetic search coil	Full amplitude (dynamic capture capable)	Degrees (3D)	Highest precision; 3D measurement; dynamic capture capability	Requires specialized equipment; coil must be embedded in prosthesis; research setting only; expensive	[23]
Infrared oculography	Dynamic tracking	% of fellow eye	Objective; quantitative comparison to fellow eye; captures dynamic movement	Specialized equipment required; may not be widely available; limited vertical measurement sensitivity	[24]
iView X eye-tracking	Dynamic tracking	% of fellow eye	Highly reproducible (ICC 0.96–0.98); commercially available; software freely available; non-invasive	Requires specific hardware; measures only horizontal motility in original validation; cost	[25]
ImageJ digital analysis	Full amplitude	mm	Free software; validated against gold standard; lower bias than smartphone apps; accessible	Requires standardized photography; post-processing time; learning curve for software	[26]
MediGrid smartphone app	Full amplitude	mm	Portable; smartphone-based; easy to use in clinic; real-time measurement	Higher limits of agreement than ImageJ; less accurate for vertical measurements	[26]
Semi-quantitative ordinal grading	Full amplitude	Ordinal scale	Simple; no equipment needed; clinically pragmatic	Coarser; rater bias possible; scale varies	[14]
Digital prosthetic eye (DPE) prototype	Full amplitude + velocity	Degrees; velocity	Captures both amplitude and velocity; potential for real-time matching to fellow eye	Experimental prototype only; not clinically available; limited by screen technology	[27]

Several strengths and limitations should be considered when interpreting these findings. The prospective design, relatively large and well-characterized cohort with substantial representation of patients both with and without orbital implants, assessment of a broad range of clinical, anatomical, and prosthetic variables, and independent photographic grading of prosthesis motility by three masked examiners with demonstrated inter-rater agreement are important strengths. However, the cross-sectional design precludes causal inference. Several socket parameters, including directional socket motility, support contour, and contracture, were based on semi-quantitative clinical assessment and are therefore subject to observer variability. Clinical measurement of fornix depth may also be influenced by examiner technique, and its reproducibility was not formally assessed. Prosthesis morphology was characterized by volume, central thickness, and posterior apex height, but overall horizontal and vertical dimensions were not recorded. Finally, although standardized photographs and three independent graders improved the consistency of the primary outcome assessment, the five-point motility scale remains semi-quantitative and does not provide the precision of instrument-based eye-tracking methods.

In summary, multivariable modeling identified socket motility, time since anophthalmia, inferior fornix depth, prosthesis alignment in primary gaze, and levator function as factors independently associated with horizontal prosthesis motility. Together, these findings suggest that visible prosthesis excursion arises from the interaction of socket movement, anatomical support, and periocular dynamics, providing a more integrated framework for understanding prosthesis motility in anophthalmic patients. A more refined understanding of these associations may also help guide future efforts to optimize clinical rehabilitation strategies, socket reconstruction, and prosthetic design to better address impaired motility, which remains one of the most persistent concerns among anophthalmic patients.

## Supplementary Information

Below is the link to the electronic supplementary material.

Supplementary file1 (DOCX 28 kb)

## Data Availability

The datasets generated and/or analysed during the current study are available from the corresponding author on reasonable request.
