# Supplementary material for: Factors associated with ocular prosthesis motility in anophthalmic patients: a multivariable analysis
Source: Int Ophthalmol. 2026 Nov 10;46(1):459. doi: 10.1007/s10792-026-04320-3 (PMC13751693; doi:10.1007/s10792-026-04320-3)
Supplement: Supplementary file 1 [file 10792_2026_4320_MOESM1_ESM.docx]

Supplement Table 1: Descriptive statistics of demographic, clinical, anatomical, and prosthesis-related baselines in 107 anophthalmic patients with artificial eyes

| **Parameter** | **Category** | **N (%) / Mean** | **Range (Interquartile range)** |
| --- | --- | --- | --- |
| Age (years) |  | 53.1±18.6 | 18,89 (37,70) |
| Time with anophthalmia (years) |  | 25.5±21.0 | 1,76 (6.5,39.5) |
| Side | Right | 47 (44%) |  |
| Gender | Male | 52 (49%) |  |
| Birthplace (immigration) | Native | 54 (51%) |  |
| Religion | Jewish | 80 (75%) |  |
|  | Muslim | 15 (15%) |  |
|  | Christian | 5 (5%) |  |
|  | Other | 5 (5%) |  |
| General health | No comorbidities | 64 (60%) |  |
| Etiology | Ocular malignancy | 50 (49%) |  |
|  | Trauma | 11 (9.9%) |  |
|  | Ocular disease | 38 (35.6%) |  |
| Orbital implant present | Yes | 46 (43%) |  |
| Additional periorbital surgeries | Yes | 14 (13%) |  |
| MRD1 (relative, mm) |  | 0.5±1.3 | -2,5 (0,1) |
| Upper eyelid position (relative) | Ptosis | 16 (15%) |  |
|  | Retraction | 1 (1%) |  |
| Levator function (mm) |  | 9.8±4.3 | 0,20 (7,12) |
| Levator function grade | Good | 71 (66%) |  |
|  | Fair | 17 (16%) |  |
|  | Poor | 15 (14%) |  |
| Lagophthalmos (mm) |  | 1.5±2.6 | 0,11.7 (0,2.3) |
| Lower eyelid laxity (relative) | Yes | 20 (19%) |  |
| Exophthalmometry (relative, mm) |  | -1.9±3.1 | -12,11 (-3,-1) |
| Prosthesis axial position (relative) | Enophthalmos | 53 (50%) |  |
|  | Exophthalmos | 8 (8%) |  |
| Inferior fornix depth (mm) |  | 7.7±2.5 | 2,21 (6,9) |
| Superior fornix depth |  | 16.4±4.5 | 1,27 (14,20) |
| Inferior fornix position (relative to inferior rim) | Anterior | 8 (7.5%) |  |
| Socket support (rounded contour) | Yes | 58 (54%) |  |
| Socket/conjunctival contracture |  | 2.7±0.6 | 1,3 (2.5,3) |
| Prosthesis motility |  | 2.9±0.9 | 1,5 (2,3.5) |
| Prosthesis primary gaze alignment | Yes | 96 (90%) |  |
| Prosthesis volume (ml) |  | 2.6±1.2 | 0.5,6 (2,3.5) |
| Prosthesis central thickness (mm) |  | 8.0±2.2 | 3,12.5 (7,9.5) |
| Posterior prosthesis height (mm) |  | 4.9±2.8 | 0,19 (3,6) |

Supplement Table 2: Blockwise multiple regression analysis for the predictive values of clinical, eyelid, socket, and prosthesis-related parameters regarding prosthesis motility examiner-rated scores, in 107 anophthalmic patients with an artificial eye

| **Motility rating**  **Blockwise regression model**  **Parameter (predictor)** | | | **Block A** Clinical Background |  | **Block B** Socket and Eyelid |  | **Block C** Socket motility |  | **Block D** Prosthesis alignment |  | **Block E** Prosthesis Structural dimensions |  | **Integrated Model (final)** |  |
| --- | --- | --- | --- | --- | --- | --- | --- | --- | --- | --- | --- | --- | --- | --- |
|  |  |  | Multiple *R^2^*=0.36  (adjusted 0.32) |  | Multiple *R^2^*=0.56  (adjusted 0.50) |  | Multiple *R^2^*=0.54  (adjusted 0.52) |  | Multiple *R^2^*=0.06  (adjusted 0.05) |  | Multiple *R^2^*=0.15  (adjusted 0.13) |  | Multiple *R^2^*=0.67  (adjusted 0.64) |  |
| **Block** | **Parameter** | **Category** | **Partial *r* (β)** | **p-value** | **Partial *r* (β)** | **p-value** | **Partial *r* (β)** | **p-value** | **Partial *r* (β)** | **p-value** | **Partial *r* (β)** | **p-value** | **Partial *r* (β)** | **p-value** |
| **A** Clinical Background | Age (Years) |  | 0.09 | 0.38 |  |  |  |  |  |  |  |  |  |  |
|  | Time with anophthalmia (Years) |  | **-0.30** | **0.006** |  |  |  |  |  |  |  |  | **-0.23** | **0.003** |
|  | Cause of anophthalmia | Trauma | 0.05 | 0.66 |  |  |  |  |  |  |  |  |  |  |
|  |  | Tumor | -0.08 | 0.51 |  |  |  |  |  |  |  |  |  |  |
|  |  | *Disease* |  |  |  |  |  |  |  |  |  |  |  |  |
|  | Orbital implant | Yes | **0.45** | **<0.0001** |  |  |  |  |  |  |  |  | 0.10 | 0.26 |
|  |  | *No* |  |  |  |  |  |  |  |  |  |  |  |  |
| **B** Socket and Eyelid | MRD1 (vs. fellow eye, mm) |  |  |  | 0.03 | 0.75 |  |  |  |  |  |  |  |  |
|  | Levator function (mm) |  |  |  | **0.31** | **0.014** |  |  |  |  |  |  | **0.15** | **0.042** |
|  | Lagophthalmos (mm) |  |  |  | -0.08 | 0.63 |  |  |  |  |  |  |  |  |
|  | Lower eyelid laxity | Yes |  |  | -0.01 | 0.95 |  |  |  |  |  |  |  |  |
|  |  | *No* |  |  |  |  |  |  |  |  |  |  |  |  |
|  | Socket support | Rounded |  |  | **0.37** | **<0.0001** |  |  |  |  |  |  | 0.05 | 0.59 |
|  |  | *Flat* |  |  |  |  |  |  |  |  |  |  |  |  |
|  | Inferior fornix depth (mm) |  |  |  | **0.19** | **0.049** |  |  |  |  |  |  | **0.19** | **0.007** |
|  | Superior fornix depth (mm) |  |  |  | 0.04 | 0.76 |  |  |  |  |  |  |  |  |
|  | Inferior fornix position (relative to inferior rim) | Anterior |  |  | -0.03 | 0.76 |  |  |  |  |  |  |  |  |
|  |  | *Posterior* |  |  |  |  |  |  |  |  |  |  |  |  |
|  | Socket/conjunctival contracture score |  |  |  | 0.09 | 0.56 |  |  |  |  |  |  |  |  |
| **C** Socket motility | Down |  |  |  |  |  | **0.30** | **0.006** |  |  |  |  | **0.20** | **0.027** |
|  | Nasal |  |  |  |  |  | 0.16 | 0.070 |  |  |  |  |  |  |
|  | Up |  |  |  |  |  | 0.10 | 0.34 |  |  |  |  |  |  |
|  | Temporal |  |  |  |  |  | **0.30** | **0.001** |  |  |  |  | **0.36** | **<0.0001** |
|  | General impression |  |  |  |  |  | 0.08 | 0.63 |  |  |  |  |  |  |
| **D** Prosthesis alignment | Prosthesis primary gaze alignment | Yes |  |  |  |  |  |  | **0.23** | **0.016** |  |  | **0.21** | **0.002** |
|  |  | *No* |  |  |  |  |  |  |  |  |  |  |  |  |
| **E** Prosthesis Structural dimensions | Central thickness (T, mm) |  |  |  |  |  |  |  |  |  | -0.17 | 0.31 |  |  |
|  | Posterior apex height (D, mm) |  |  |  |  |  |  |  |  |  | 0.20 | 0.18 |  |  |
|  | Posterior projection index (D/T) |  |  |  |  |  |  |  |  |  | 0.27 | 0.94 |  |  |

Values in boldface mark a significant independent predictive effect of the parameter (<0.05) in the regression (general linear)
Categories in italics mark the reference category
